# Statistics Regarding Tuberculosis in the Prussian Slaughter-houses

**Published:** 1898-08

**Authors:** 


Statistics Regarding Tuberculosis in the Prussian Slaughter-houses. According to the report of the Minister of Agriculture and Forestry the percentage of slaughtered animals affected with tuberculosis varied in the Prussian slaughter-houses from January 1st until December 31, 1896, between 0.9 per cent. and 39.5 per cent.

The district of Osnabrück showed the smallest percentage of 0.9; then follow Gumburnen, with 3.1 per cent.; Minden, with 3.6 per cent.; Cologne, with 3.6 per cent.; and Siegmaungen, with

4.2 per cent.; Cassel showed 5.9 per cent.; Hanover, 6.7 per cent.; Munster, 7.1 per cent.; Breslau, 8.4 per cent.; Posen, 9.4 per cent., and Stade 9.8 per cent. A percentage of between 10 and 20 per cent. was distributed over the districts as follows: Maneinwerder, 12.5 per cent.; Berlin, 15 1 per cent.; Potsdam, 15.6 per cent.; Frankfurt on the Oder, 13.2 per cent.; Oppeln, 13.3 per cent.; Arnsberg, 13.5 per cent.; Wiesbaden, 13.6 per cent.; Stettin, 15.4 per cent.; Leignitz, 10.3 per cent.; Magdeburg, 19.3 per cent.; Erfurt, 12.0 per cent.; Hildesheim, 11.3 per cent.; Aurich, 16.1 per cent.; Coblentz, 15.9 per cent., and Treves 10.9 per cent. The following districts showed over 20 per cent.: Dantzig, 26.9 per cent.; Koslin, 21.4 per cent.; Stralsund, 28.9 per cent.; Bromberg, 20.9 per cent.; Merseburg, 26.3 per cent.; Schleswig, 39.5 per cent.; Luneburg, 20.6 per cent, and Aix-la-Chapelle, 25.1 per cent. Of 812,731 animals slaughtered in Prussia, or at least imported in a slaughtered condition, 107,214, or 13.2 per cent., were found to be affected with tuberculosis.

